# Sheehan's syndrome misdiagnosed as encephalitis: A case report and literature review

**DOI:** 10.1002/ibra.12096

**Published:** 2023-03-20

**Authors:** Xiao‐Yan Yang, Yong‐Su Zheng, Jin‐Mei Tuo, Hai‐Qing Zhang, Zu‐Cai Xu

**Affiliations:** ^1^ Department of Neurology Affiliated Hospital of Zunyi Medical University Zunyi China; ^2^ Department of Health Promotion System Sciences, Division of Health Sciences, Graduate School of Medicine Osaka University Suita Japan

**Keywords:** encephalitis, hypopituitarism, postpartum hemorrhage, Sheehan's syndrome

## Abstract

A 48‐year‐old female patient was hospitalized for 5 days after a cold. Encephalitis was considered after preliminary history and routine examination, but the patient did not show significant improvement after antiviral treatment. At this time, magnetic resonance imaging indicated pituitary atrophy, and the patient's medical history was assessed. She had a history of postpartum bleeding and amenorrhea 15 years ago. The supplementary examination indicated hormonal abnormalities. These suggested that the patient may have had Sheehan's syndrome (SS). After hormone supplementation treatment for 10 days, her condition improved. This case suggested that in female patients with neuropsychiatric disorders with a history of previous postpartum hemorrhage, attention needs to be paid to screening for SS to improve the related diagnosis and treatment rate.

## Graphical abstract

A case of Sheehan's syndrome misdiagnosed as encephalitis was reported and the related literature about the disease was reviewed.

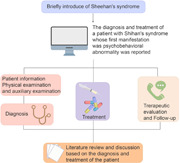

## 1 INTRODUCTION

Sheehan's syndrome (SS) is a neuroendocrine disease caused by severe postpartum hemorrhage leading to ischemic pituitary necrosis.^1^, ^2^, ^3^ According to epidemiological reports, this is an important reason for hypopituitarism in underdeveloped and developing countries.^2^ The prevalence rate in women over 20 years old is about 3.2%.^4^ Because of its low incidence rate and different onset times, there is no relevant standard for regularly detecting pituitary‐related hormones in patients with postpartum hemorrhage. The typical symptoms of viral encephalitis are mainly mental and consciousness disorders. However, patients with SS may have mental and behavioral abnormalities as the disease develops.^5^ In this paper, a case of SS misdiagnosed as encephalitis due to reported cognitive behavior abnormality is reported, and the literature is reviewed.

## 2 PATIENT INFORMATION

The female patient, 48 years old, was admitted to the Department of Neurology on February 23, 2019, for “5 days because of abnormal mental behavior.” Five days before admission, the patient has mental disorder after catching a cold, characterized by gibberish, self‐talk, involuntary movement of the hands and feet, hallucinations, and auditory hallucinations. After 2 days, the patient's symptoms changed to silence. She did not communicate with family members, did not open her eyes, she had no fever, headache, cough, expectoration, dyspnea, or convulsion or consciousness disorder. For further diagnosis and treatment in the emergency department of our hospital, the patient was admitted to the department of neurology. Since the onset of the disease, her mental state was poor, her diet was reduced and she had insomnia, no fecal incontinence, and no significant weight loss. Menstrual and reproductive history: The patient had a history of postpartum hemorrhage 15 years ago. In addition, she had postpartum lactation failure and amenorrhea, three pregnancies, and three deliveries. On examination at admission, the temperature was 37°C, the pulse rate was 73 times per minute, the respiratory rate was 22 times per minute, and blood pressure was 118/56 mmHg. Physical examination of digestive system, circulatory system and respiratory system is normal. This research was approved by the Ethics Committee of the Affiliated Hospital of Zunyi Medical University (the Ethical Approval Number: KLL‐2022‐812).

## 3 PHYSICAL EXAMINATION AND AUXILIARY EXAMINATION

The patient was silent and did not cooperate with the physical examination. The meningeal stimulation sign was negative. The diameter of the pupil was about 3 mm. The photosensity, tendon reflex, physiological reflex and pathological sign was negative other physical examinations cannot be completed because the patient did not cooperate with the examination. The rest can not be identified.

Auxiliary examination upon admission indicated no abnormality on performing emergency computed tomography (CT). The liver function test showed that aspartate aminotransferase was 40 U/L. The rest of the parameters were normal. The renal function test showed 87 µmol/L uric acid and 0.58 mg/L serum cystatin C. The rest of the parameters were normal. Blood sugar was normal. Electrolytes and blood lipids were abnormal (Table 1).

**Table 1:** Changes of electrolyte and blood lipids in serum.

Inspection items	Normal reference value	Results of admission	Results from discharge
Sodium	137–147 mmol/L	124.51 mmol/L ↓	137.9 mmol/L
Chlorine	99–110 mmol/L	93.7 mmol/L ↓	107.9 mmol/L
Magnesium	0.67–1.04 mmol/L	1.05 mmol/L	1.03 mmol/L
Triglyceride	<1.7 mmol/L	2.72 mmol/L ↑	2.16 mmol/L
Total cholesterol	<5.2 mmol/L	8.55 mmol/L ↑	6.44 mmol/L
LDL cholesterol	<3.12 mmol/L	5.71 mmol/L ↑	4.35 mmol/L
Apolipoprotein B	0.55–1.30 g/L	1.62 g/L ↑	1.32 g/L

## 4 DIAGNOSIS AND TREATMENT PROCESS

**Figure 1:**
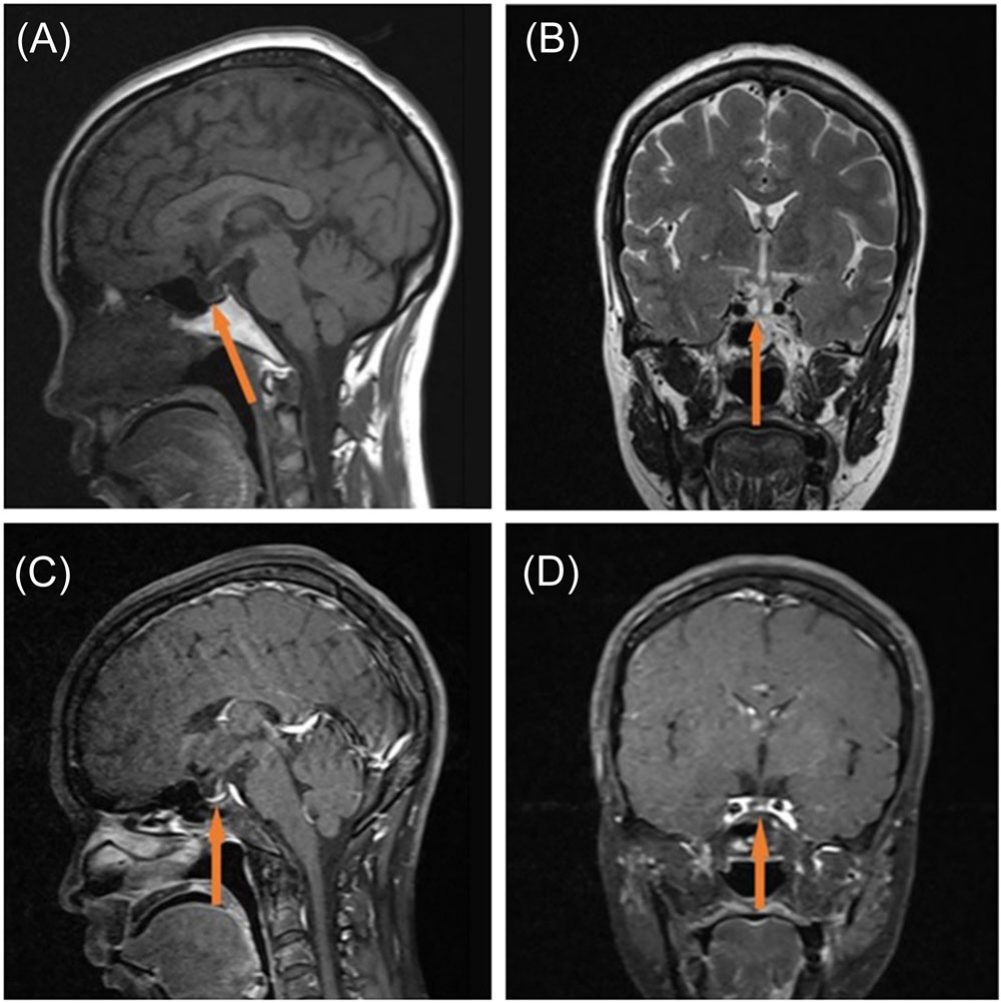
Nuclear magnetic resonance imaging manifestations of the patient indicate pituitary atrophy (orange bold arrow): (A) sagitta T1 weighted, (B) coronal T2 weighted, (C) sagitta T1‐weighted postcontrast, and (D) coronal T1‐weighted coronal. [Color figure can be viewed at wileyonlinelibrary.com]

Combined with the medical history, the disease was considered to be viral encephalitis. A lumbar puncture should have been performed for a cerebrospinal fluid (CSF) examination, but the patient's family refused. Antiviral therapy was administered, but the therapeutic effect was not ideal. On the third day after the admission, plain and enhanced nuclear magnetic resonance imaging (MRI) of the pituitary indicated that the pituitary gland was flattened, with a uniform signal. The maximum height was 0.1 cm. No abnormality was found in the position of the pituitary stalk. The enhanced scan showed that the pituitary gland was enhanced. No abnormality was found in the size and shape of the sella turcica. Because of the sella turica vacuole and pituitary atrophy, the signal at the sella turcica is water‐like signal (Figure 1). Chest CT examination showed a small amount of fibrosis. There were no obvious abnormalities on the electrocardiogram (ECG). The pituitary‐related hormone examination was abnormal (Table 2).

**Table 2:** Baseline hormone profile.

Parameters examined	Normal reference value	Inspection result
FT3	2.77–6.31 pmol/L	1.6 pmol/L ↓
FT4	10.45–24.38 pmol/L	5.9 pmol/L ↓
TSH	0.5–4.8 µLU/ml	2.767 µLU/ml
Triiodothyronine	1.02–2.96 nmol/L	0.24 nmol/L ↓
Thyroxine	55.47–161.25 nmol/L	18.3 nmol/L ↓
Thyroid peroxidase antibody	0–34 IU/L	475.7 IU/L ↑
TGA	0–115 IU/L	542.8 IU/L
Thyroglobulin	3.5–77 ng/ml	0.917 ng/ml ↓
HGH	0.126–9.88 ng/ml	0.07 ng/ml ↓
Sex hormone binding globulin	32.4–128 nmol/L	138 nmol/L ↑
Free testosterone index	0.297–5.62	0.06 ↓
Total testosterone	0.3–1.6	0.087 ↓
Cortisol	172–497.4 nmol/L	48.5 nmol/L↑

Abbreviations: FT3, serum free tri‐iodothyronine; FT4, serum free thyroxine; HGH, growth hormone; TGA, thyroglobulin antibody; TSH, thyrotropin.

The routine blood test showed that the total number of red blood cells was 2.99 × 10^12^/L. Hemoglobin was 92 g/L. Hematocrit was 0.27 L/L. The total platelets were 95 × 10^9^/L. The platelet‐specific volume was 0.11%. Assessment of medical history indicated that the patient had a history of postpartum hemorrhage 15 years ago. In addition, she had postpartum lactation failure, amenorrhea, three pregnancies, and three deliveries. Combined with the patient's postpartum hemorrhage history and related examination results, she received treatment with prednisone 5 mg in the morning and 2.5 mg in the evening. Levothyroxine 25 μg was taken orally once a day. The dosage was changed to 50 μg after 10 days, and then to 75 μg after 20 days. After 3 days of treatment, the patient's mental disorder improved, after 10 days of treatment, the electrolyte and blood lipid of the patient significantly improved on re‐examination (Table 1). Her mental symptoms improved significantly, so she was discharged from the hospital. She was instructed to attend follow‐up at the clinic after 1 month. After 1 month, the patient recovered to normal state, her pituitary hormone levels, electrolytes, and blood lipids were normal. Since then, the patient has been attending follow‐up at the endocrine clinic. One year after discharge, we conducted a follow‐up call with her. The patient said that she had no symptoms and could take care of herself.

## 5 DISCUSSION

SS is a series of clinical symptoms of hypopituitarism caused by adenohypophysis necrosis due to massive hemorrhage during or after delivery.^3^, ^5^ It mainly involves the thyroid, adrenal, gonad, and other target glands of hypothyroidism.^6^, ^7^ The main clinical manifestations are (1) insufficient gonadotropin: with amenorrhea, postpartum nonlactation, infertility, vaginal dryness, and anemia^8^, ^9^; (2) insufficient secretion of thyrotropin: it is characterized by chilliness, rough skin, sparse hair, and fatigue. Some patients may have systemic myxedema and apathetic expression^10^; (3) growth hormone deficiency: abdominal obesity, reduced attention, and memory^11^, ^12^; and (4) insufficient adrenocorticotropic hormone secretion: the symptoms include hypotension, hypoglycemia, fatigue, nausea, and vomiting. Because of its complex clinical manifestations, hidden onset, and lack of specificity, patients cannot actively identify the main symptoms. Hence, it is easy to cause clinicians to give wrong diagnosis. A common clinical manifestation of viral encephalitis is mental disorders.^13^ The patient, in this case, was admitted to the neurology department due to abnormal mental behavior and had a history of a cold before the illness. Therefore, the patient was initially diagnosed as viral encephalitis. Antiviral therapy was administered, but the therapeutic effect was not ideal.

Since the head MRI scan showed pituitary abnormalities, further pituitary hormone detection was performed. The results of the hormone examination indicated decreased pituitary function. On assessment of the medical history, the patient reported a history of postpartum hemorrhage. The reduction of pubic hair and axillary hair was found by further physical examination. The diagnosis of SS was finally confirmed through consultation with the endocrine department. Because of the abnormal mental behavior of the patient, it was considered that the secondary adrenocortical dysfunction caused by pituitary atrophy leads to electrolyte disorder, repeated hyponatremia and cell edema, which cause mental disorders in patients.^14^, ^15^, ^16^ In addition, mental symptoms in patients with SS may also be caused due to the following reasons: (1) the decrease of glucocorticoid leads to a decrease of blood glucose, which leads to necrosis of cerebral cortex cells and neurotransmitter transmission disorders. It often manifests in clinical symptoms such as lethargy, apathy, and limb twitching; (2) Hypothyroidism leads to decreased cerebral blood flow and cerebral cell ischemia and hypoxia, which will lead to untypical symptoms, such as silence and fear.^17^, ^18^; (3) Decreased gonadal function leads to insufficient secretion of sex hormones, which weakens the protection of the nervous system.^19^ It makes some patients have insomnia, anxiety, and depression.^20^, ^21^ In addition, hypotension causes insufficient blood volume circulation, leading to necrosis of brain cells due to ischemia and hypoxia.^22^, ^23^ The literature has also reported that the abnormal increase of the C‐fos gene in the brain during hypotension leads to the disorder of neural signal transmission,^24^ which leads to coma and lethargy. It is also one of the causes of mental disorders. The treatment of this disease is hormone replacement therapy. Rational drug use is not only important to correct endocrine disorders but also important to reduce the mortality caused by hypopituitarism. When patients have secondary hypothyroidism and adrenal cortical dysfunction, glucocorticoids should be used before thyroid hormone replacement, and hormone replacement therapy should be used when gonadotropin deficiency and hypogonadism occur.^25^

Notably, some studies have shown that with glucocorticoid supplementation, the use of large doses of glucocorticoid will cause apoptosis of nerve cells and atrophy of the hippocampus, leading to mental disorders.^26^ In addition, it can selectively inhibit neural electrical activity, destroy the balance between neurotransmitters, and thus cause mental disorders.^20^ Therefore, the onset and treatment of SS can induce mental disorders. Mental disorders may occur in the clinical manifestations and treatment of Sean syndrome, which requires the attention of clinicians.

## 6 CONCLUSION

As a result, because of its long course and a typical clinical manifestations and the lack of understanding about the disease for young doctors, SS is easy to be misdiagnosed when the patient's clinical manifestations are mental disorders. For female patients with mental disorders who visit the department of neurology, clinicians must master her detailed medical history, fertility history and symptoms related to the nervous system.

If necessary, relevant examinations should be carried out to diagnose and treat the disease in a timely manner.

## AUTHOR CONTRIBUTIONS

**Xiao‐Yan Yang and Yong‐Su Zheng**: Writing‐original draft. **Hai‐Qing Zhang and Jin‐Mei Tuo**: Clinical data collection. **Zu‐Cai Xu**: Conceptualization, supervision, writing‐review & editing. All authors reviewed the manuscript. All authors read, revised, and approved the final manuscript.

## CONFLICT OF INTEREST STATEMENT

The authors declare no conflict of interest.

## ETHICS STATEMENT

This research was approved by the Ethics Committee of the Affiliated Hospital of Zunyi Medical University (the Ethical Approval Number: KLL‐2022‐812). This study is a retrospective study and will not cause adverse consequences to patirnts, so it is uncenessary to sign an informed consent form for this study.

## Data Availability

The authors confirm that the data of this study are available within the article.
